# STEM CELLS HEMATOPOIETIC NICHES AND INFLAMMATORY RESPONSE TO DIFFERENT SYNTHETIC PROSTHESIS IMPLANTED IN RAT WITH INCISIONAL HERNIAS

**DOI:** 10.1590/0102-6720201700020007

**Published:** 2017

**Authors:** Renato Lamounier BARBIERI, Suely de Fátima PARREIRA, Sarah do Valle STUDART, Aline Riquena DA-SILVA, Ivone da Silva DUARTE, Pedro Luiz Squilacci LEME

**Affiliations:** 1 Medical School, Nove de Julho University, São Paulo, SP, Brazil

**Keywords:** Abdominal wall, Hernia, ventral, Hematopoietic stem cells, Surgical mesh, Adverse effects

## Abstract

**Background:**

Extramedullary hematopoiesis depends on complex pathophysiological mechanisms linked to hematopoietic stem cells and the proteins considered mediators of the inflammation. The identification of hematopoietic cells outside bone marrow in the adult is an occurrence that can occasionally follows the inflammatory response, was considered a secondary occurrence, but current biomolecular studies have changed that concept.

**Aim:**

Describe the presence of clusters of precursor cells of platelets (megakaryocytes), and cells of the inflammatory response in the abdominal wall and spleen of rats with experimentally induced incisional hernias and repaired with different synthetic prostheses.

**Methods:**

Twenty-five rats with incisional hernias previously performed, were divided into groups of five animals each: Group 1, repair of the hernia defect without prosthetic implant; Group 2, repair with polypropylene prosthesis; Group 3, repair using polypropylene with low weight; Group 4, the use of polypropylene and polyglecaprone prosthesis; Group 5, of polypropylene and polyglactin prosthesis. All prostheses were cut in rhombus format with area 2,625 cm². The animals were reoperated after 10 days, the abdominal walls were removed with the viscera attached to them and the material was processed for histological study.

**Results:**

Megakaryocyte niches in the abdominal wall and spleen, occasionally removed together with the adhesions produced in animals with implantation of prostheses and significant inflammatory reaction.

**Conclusion:**

The intense inflammatory reaction due to the prostheses with polypropylene in their composition was disproportionate to the expected response, indicating that further studies should be accomplished including immunophenotyping evaluation and specific panels of monoclonal antibodies to better understand the findings.

## RESUMO

**Racional:**

A hematopoiese extramedular depende de mecanismos fisiopatológicos complexos, havendo relação destas células-tronco hematopoiéticas com proteínas mediadoras da inflamação. A identificação de células hematopoiéticas fora da medula óssea no adulto, situação que ocasionalmente pode acompanhar a resposta inflamatória era considerada ocorrência secundária, mas estudos biomoleculares modificaram este conceito.

**Objetivo:**

Descrever agrupamentos de células precursoras das plaquetas (megacariócitos) e células da resposta inflamatória, na parede abdominal e no baço de ratos com hérnias incisionais induzidas experimentalmente e reparadas com diferentes próteses sintéticas.

**Métodos:**

Vinte e cinco ratos com hérnias incisionais previamente realizadas foram distribuídos em grupos com cinco animais: Grupo 1, reparo do defeito herniário sem implante de prótese; Grupo 2, reparo com prótese de polipropileno; Grupo 3, reparo empregando polipropileno com baixa gramatura; Grupo 4, utilização de prótese de polipropileno e poliglecaprone; Grupo 5, prótese de polipropileno e poliglactina. Todas as próteses foram recortadas na forma de losangos com área de 2,625 cm². Os animais foram reoperados após 10 dias, as paredes abdominais foram retiradas em bloco com as vísceras a elas aderidas e o material foi processado em rotina histológica.

**Resultados:**

Foram evidenciados nichos de megacariócitos na parede abdominal e no baço coletado juntamente com as aderências em animais com implante de próteses, além de reação inflamatória significativa.

**Conclusão:**

A intensa reação inflamatória, local e sistêmica em relação às próteses com polipropileno em sua composição, foi desproporcional à resposta esperada, requerendo aprofundamento do estudo com avaliação da imunofenotipagem e painéis específicos de anticorpos monoclonais para melhor esclarecimento.

## INTRODUCTION

During intrauterine life, hemangioblasts from vitellin sac mesoderm present great capacity of hematopoietic and vascular differentiation, originating the first blood vessels and hemangioblasts, due to embryo needs for nutrients and oxygen from maternal circulation. Mesenchymal cells (angioblasts) form blood islets and the spaces inside these islets origin a primitive endothelium with cells that will differentiate into blood cells; this phenomenon begins in the liver, and after in the spleen, bone marrow and lymphnodes. Although the first blood cells and plasm have their origin from vessels located in the vitellin sac and allantoids, blood is only identified after the second month of the embryonic development^6^.

There, can be found hematopoietic cells in the vitellin sac with great differentiation capacity, like monocyte and megakaryocyte precursor cells. After birth and in normal condition, erythrocyte, granulocyte and platelet hematopoiesis occurs in bone marrow; lymphocytes are mainly formed in lymphoid tissue (lymphnodes, spleen, thymus, and mucosa lymphoid tissue), but this function can be assumed by other organs, such as in situations following benign as well as malignant disorders^10^ ^,^ ^13^ ^,^ ^14^ ^,^ ^21^.

The occurrence of blood cells out of the bone marrow in adult is defined as extramedullary hematopoiesis, that can form nodes inclusively identified during image routine scan^17^ ^,^ ^18^. This situation that formerly was considered to be secondary and of minor importance, according to biomolecular assay is not now established as incidental, raising the stem cell concept in its microenvironment (stem cells niches). The development of these cells in other tissues than bone marrow implies on hematopoiesis embryonic patterns, with the reactivation of stem cells niches and participation of extracellular matrix, of stromal cells as well as chemokines representing an complex process of organic response due to adverse situation yet to be fully understood in mammals^8^ ^,^ ^10^ ^,^ ^13^ ^,^ ^26^ ^,^ ^30^.

Inflammatory process and immune response that occur in tissue repairing, with the presence of antigenic and phagocytic cells, are triggering factors, yet occurring all blood cells hematopoiesis mainly in spleen and liver. The phenomenon that accompanies inflammatory process initiates in microcirculation firstly due to chemical mediators that act over arterioles as well as secondary chemical mediators released during cellular inflammation response. The opening of capillary sphincters by catabolism, hydrogen ion and chemical mediators increase vascular permeability and hyperemia, with plasmatic albumin rich fluid flow, resulting in hemoconcentration, erythrocyte aggregation, increase of blood viscosity and local hypoxia. Endothelium also goes through changes, occurring platelet aggregation and cloth formation. In acute phase, leukocyte response initiates and these cells are observed on vessel wall margins and stasis in circulation leads to the red blood cell exit from vascular bed, occurring diapedesis and neutrophil migration to the inflammatory connective tissue^10^ ^,^ ^14^ ^,^ ^20^ ^,^ ^28^.

Repairing of extended incisional hernia implies on the dissection of extensive areas, compromising significantly fibrotic and repairing tissues, as well as in the use of synthetically made prosthesis. Polypropylene is used since Usher pioneer work in 1958 and several options have been developed aiming at reducing inflammatory response to the implantation, like the diminishing used grammature of used material or the association of a smaller polypropylene framework to other absorbing material. However, triggered inflammatory response is significant, even with different materials available nowadays^2^ ^,^ ^3^ ^,^ ^11^ ^,^ ^16^ ^,^ ^22^ ^,^ ^25^ ^,^ ^29^.

The aim of this study was to describe the occurrence of groups of precursor cells of platelets (megakaryocytes) as well as some of other cells of the inflammatory response, both in the abdominal wall and spleen of rats with experimentally induced incisional hernias and repaired with different synthetic prostheses.

## METHODS

This experiment was conducted at the University Nove de Julho, São Paulo, Brazil, after authorization by the Commission of Ethics in the Use of Animals (Protocol AN 0034/13). The general rules for experimental research in Advanced Surgical Skills Laboratory of the institution are strictly supervised and comply with the precepts of “rational use of animals for experimentation”^15^; in compliance with Federal Law No. 11,794 October 2008 and Decree No. 6,689, July 2009. All animals received anesthetic induction before surgical procedures and before death, as well as general care like feeding, antibiotic therapy and standardized analgesia for postoperative period.

Twenty-five Wistar rats (*Rattus Norvegicus, var. Albinus, Rodentia, Mammalia*), which are genetically similar and present not only isogenic line, but reliable results in studies with small numbers of animals^4^ ^,^ ^24^ were randomly distributed in five groups with five animals each; before surgical interventions they were kept in individual cages with access to standardized food and water ad libitum, at 25° C room temperature and a 12 h photoperiod. The animals, with an average weight of 339 g were anesthetized with intraperitoneal injection of ketamine hydrochloride (80 mg/kg) and xylazine (10 mg/kg), being held a median incision with 4 cm in length, opening of the abdominal cavity in linea alba measuring 3.5 cm and a suture in the middle third of each side of the incision was made, with the eversion of the edges of the abdominal rectum muscle without encompassing the peritoneum, thus creating a defect with 3.5x1.5 cm. The first phase of the experiment ended with nylon suture 5-0 only skin of animals, which were returned to the cages and observed for 10 days.

**FIGURE 1:**
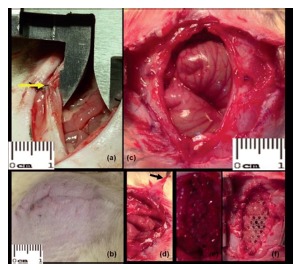
First phase of the experiment: A) eversion point (arrow) in the middle third of the incision, without encompassing the peritoneum, to perform the hernia defect; B and C) developed hernia after ten days of observation; D) hernia sac (arrow). Second phase of the experiment: E and F) different prostheses sutured to the edges of the hernia defect on the 10th day after the first operation.

On the 10^th^ day after the initial operation they were again operated to evaluate incisional hernia and adhesions formed initially^2^ ^,^ ^25^. Undone these adhesions, in each of the five groups one of the following procedures was held: Group 1, simple approximation of the edges of the hernia opening by simple stitches using polyglactin 910 number 5-0 without prosthetic implant; in Group 2 the defect was repaired with hernia polypropylene prosthesis sutured to the edges, an option considered to be reference to the greater inflammatory reaction expected; in Group 3 polypropylene with low weight was employed; in Group 4, polypropylene prosthesis and poliglecaprone; and in Group 5 polypropylene and polyglactin. The four different synthetic prostheses were donated to avoid conflicts of interest, cut in the form of rhombus with 3.5x1.5 cm, corresponding to an area of 2.625 cm² and fixed to the edges of the holes with polyglactin 910. After the procedures proposed for each group, the surgical procedures were completed with skin suture using nylon thread 5-0 and the animals were observed for 10 more days (Figure 1).

After a period of 10 days, the animals were again operated for withdrawal of previous abdominal walls, with any organs adhered to prostheses, allowing the macroscopic evaluation. The pieces obtained were preserved in 10% formalin for 24 h and then dehydrated with ethanol in progressive concentration, diaphanizated with xylene and included in paraffin; 5 µm serial sections were made and stained with H&E. The resulting slides were photographed with the aid of optical microscope with four objectives (10, 20 and 40), as well as 10x eyepiece, providing a detailed resolution (40, 100, 200 and 400 x), equivalent to 20, 50, 100 and 200 µm respectively.

## RESULTS

**FIGURE 2:**
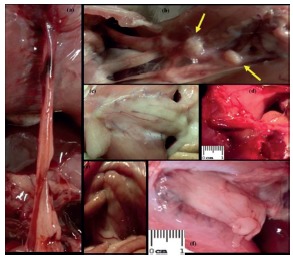
Reoperation ten days after repair of the defects with: A) Group 1 (no prosthesis): adhesion of the omentum to the incision; B and C) Group 2 (polypropylene): foreign body granuloma areas (arrows) and adhesions of the greater omentum; D) Group 3 (low weight polypropylene): intense vascularization and firm adhesions on the prosthesis; E) Group 4 (polypropylene and poliglecaprone): small bowel adhesions; F) Group 5 (polypropylene and polyglactin): graft covered by the greater omentum.

There were adhesions from different abdominal structures to the sutured wall and to the implanted prosthesis to all groups, as well as inflammatory reaction suggested by intense vascularization and macroscopic evidenced granuloma, also evidenced on reoperation held after 10 days for block withdrawal of the abdominal wall and organs and adhered structures (Figure 2).

**FIGURE 3:**
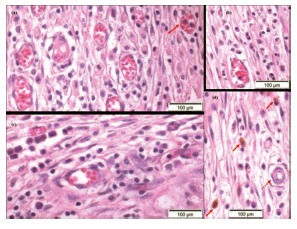
A, B and C) Great amount of congested blood vessels, granulocytes in lumen of a vessel (arrow) and inflammatory infiltrate; D) phagocyted hemosiderin, allowing the identification of macrophages (arrow heads) and arteriole (dashed arrow) H&E, 200 x.

The histological analysis showed intense cellular response in the connective tissue around the implant site, neovascularization and vessels with many red blood cells in luminal space (Figure 3). Morphologically, the lumen of vessels is surrounded by an endothelial layer, venules are similar to capillaries in diameter, but with some muscle cells in their walls and in typical arterioles, the thickness of the layer of smooth muscle cells is similar to the diameter of the vessel. These structures were found in several microscopic fields, as well as undifferentiated perivascular mesenchymal cells (pericytes), which can differentiate from other cells of the connective tissue, with their elongated nuclei and dense chromatin, especially in adjacent areas of capillaries.

**FIGURE 4:**
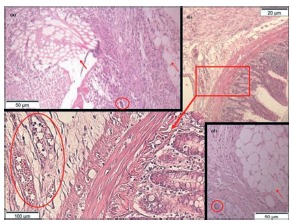
Histological sections stained by H&E with adhesions and inflammatory reaction on the implanted material: A) 100x, where the polypropylene prosthesis was located (arrow), adhesion of the greater omentum (dashed arrow) and giant cell foreign body (detail); B) 40x and C) featured -200 x, adhesion of the small intestine to the abdominal wall, congested vessels with granulocytes (detail); D) 100x, adhesion of the greater omentum (dashed arrow) to the abdominal wall and giant cell foreign body (detail).

Firm adhesions between the greater omentum, small intestine, spleen and liver and the abdominal wall and implanted prostheses were found frequently. There had been intense migration of granulocytes, polymorphonuclear eosinophil being evidenced with acidophil cytoplasm granules; typical cells with scant cytoplasm, large rounded and dark; plasma cells, which produce antibodies, denoting a significant immune response, with characteristic and eccentric rounded nuclei. Several macrophages with cytoplasm-phagocytized hemosiderin, suggesting bleeding next to the site, as well as many foreign body giant cells phagocytizing inert material and cellular debris were identified (Figures 3 and 4).

**FIGURE 5:**
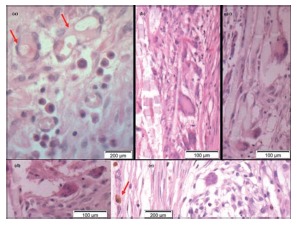
Histological sections stained by H&E with inflammatory reaction to the implanted material: A) 400x, intense inflammatory response and giant cells internalizing inert material (arrows); B, C and D) 200x, granulomatous reaction to the implanted material, with foreign body giant cells; E) 400x, inflammatory reaction, and giant cell and phagocytized hemosiderin in a macrophage cytoplasm (arrow).

Granulomatous areas can be observed in some chronic inflammation, being formed by grouping of defense cells and occasionally might be identified macroscopically (Figure 2). They are mainly composed by lymphocytes, plasma cells, histiocytes and monocytes, many giant cells, with or without necrosis sign. Foreign body granuloma can develop for response to an agent of low immunogenic activity, as it occurs with the inert non-digestible materials. Many Langerhans cells, with peripheral nuclei arranged in a horseshoe shape were identified (Figure 5).

**FIGURE 6:**
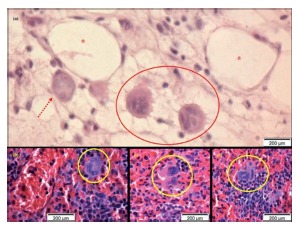
Histological sections stained by H&E 400x: A) giant cell (dashed arrow) and niche with platelet precursor cells (megakaryocytes), with lobed nuclei and cytoplasmic extensions (featured), next to the lymphatic vessels (*) of the greater omentum, acceded to the abdominal wall of animal with polypropylene prosthesis implantation of low grammage; B, C and D) megakaryocytes (highlighted) in spleens, inferring systemic stimulation of material implanted in the abdominal wall.

Cells with histological features of megakaryocytes forming niches were identified in the greater omentum and spleen, eventually adhered to the abdominal wall, which were also processed for histological study (Figure 6). The presence of these hematopoietic cells outside the bone marrow and the patterns of embryonic hematopoiesis in spleen infer the intensity of the inflammatory reaction observed in this experiment.

## DISCUSSION

Connective tissues, which are originated from mesenchyme, are rich in blood vessels, phagocytic and antibody-producing cells, as well as elastic and collagen fibers; their most common cells are fibroblasts (cells) and fibrocytes (mature cells). In normal connective tissue there can also be seen lymphocytes, eosinophils, mesenchymal undifferentiated and slightly differentiated cells that can turn into plasma cells, and macrophages (called histiocytes when tissue resident), with phagocytes aggressive agents, cellular debris, inert particles and which may form foreign body giant cells^3^ ^,^ ^12^ ^,^ ^27^.

Exudative phenomena are characteristic to inflammation, accompanied by increased vascular permeability, swelling, inflammation and the release of chemical mediators; neutrophils are the first cells to migrate to inflammatory focus, favoring the release of secondary chemical mediators; as the alteration continues, fibroblasts and capillary endothelial cells also try to repair the damage. Phagocytosis is one of the most important local and systemic defense mechanism, and when the condition becomes chronic, macrophages increase their cytoplasmic volume, with high phagocytic activity, and in chronic granulomatous inflammation they tend to huddle together, acquiring epithelioid aspect thus forming plurinucleated giant cells^3^ ^,^ ^5^ ^,^ ^6^ ^,^ ^27^ ^,^ ^28^.

Monocyte migrated from blood gives rise to macrophages in inflammatory focus, especially when there is granulation tissue. The action of fibroblasts is critical for tissue repair and vascular - connective neoformation is characteristic of chronic processes. Plasma cells are differentiated from B lymphocytes and monocytes increase phagocyte activity by stimulation of T lymphocytes, forming compact cell groups^12^ ^,^ ^19^.

Langerhans cells have small nuclei, are similar in their horseshoe like form, and can reach up to 100 µm in diameter; when formed in response to non-digestible foreign bodies, they show irregular contour, and can achieve approximately 300 µm^12^ ^,^ ^19^ ^,^ ^26^ ^,^ ^28^. There were evidenced macroscopically granuloma and found many cells with such features in this study.

The hypersensitivity to a non-degradable product results from a complex relationship between antigens of the foreign element to the organism, prolonged activation of macrophages, T helper cell response and hyperactivity of the B cells, circulating immune complexes and biological chemical mediators, with intense activity of enzymes, cytokines, expression of MHC complexes and Th1 CD4 + lymphocytes; these cells induce the release of interleukin-1 and macrophage chemotactic factors, resulting in granuloma^3^ ^,^ ^12^ ^,^ ^19^ ^,^ ^24^ ^,^ ^26^ ^,^ ^28^.

Signs of intense inflammatory reaction were evidenced in all studied animals, which presented granulation tissue, various degrees of angiogenesis and fibrosis. Blood vessels with a thin endothelial layer and replete by red blood cells, support connective tissue with great density of fibroblast accompanied by buildup of several granuloma, denoting a disproportional inflammatory response than the expected to the synthetic implanted material were observed.

The omentum is formed by a trabecular structure, with blood vessels and adipose tissues, being considered in the available literature an endocrine citocine secretor organ with great functional capacity. It is classified by the structure as a lymphatic reticular organ, rich of arterioles, venules, lymphocytes, mesothelial and endothelial cells, macrophages, monocytes, granulocytes, fibroblasts and adipocytes, possessing angiogenic capacity; stem cells have already been isolated from this tissue, classically associated to abdominal defense against infection or prevention of some complication caused by adhesion induced by surgical manipulation or synthetically material implanted^2^ ^,^ ^3^ ^,^ ^23^ ^,^ ^25^. The omentum was the abdominal structure that most covered the implant material used, being conducive to deployment of megakaryocytes niche identified in the study here described.

The spleen is an organ lymphoid brought into the bloodstream and presents a capsule of dense connective tissue that forms trabeculae and has scant smooth muscle fibers; in its interior primarily monocytes and lymphocytes are formed, as well as their phagocytic cells act against aggressive agents, even being inert, that accumulate in their macrophages.

Reticular fibers are important to the organ constitution, divided into white pulp that contains lymphocytes in different maturation stages, and red pulp formed by Billroth cords, sinusoids (splenic sinuses) full of blood and covered by elongated phagocytic cells that perform hemocatheresis absorbing fragmented red blood cells^26^.

Reticular cells, macrophages and lymphocytes occupy the stream of lymphoid tissue between red pulp and lymphatic nodules, the region where antigen that triggers immune response is retained; in Billroth cords, there can be evidenced primitive reticular cells, resident and visitors macrophages, lymphocytes, plasma cells, red blood cells, platelets and granulocytes. Branches of the splenic artery are covered by a layer formed by T cells that are expressed forming nodules where B cells are dominant. Terminal arterioles reach the red pulp and in their capillaries endothelium and basal lamina, phagocytic cells, adventitial cells (mesenchymal undifferentiated cells), as well as lymphocytes, presenting high activity in the immune response can be identified^6, 19, 26^.

The spleen has a hematopoietic function in human fetuses after half pregnancy term, persisting certain residual activity in the neonatal period, which soon disappears. The megakaryocyte arises at early embryonic period in the liver, spleen and thymus; its number decreases after half the period of intrauterine life, but persists for a few weeks after birth, when the spleen starts to produce mainly the red blood cells^6.13^. In normal adult spleen, there can be found mature hematopoietic elements which are derived from circulating stem cells. These, in this organ, will complement differentiation^21^.

Bone marrow contains many cells derived from mesenchymal stem cells, represented by fibroblasts, adipocytes, chondrocytes, and osteoblasts, endothelial cells and vascular, including perivascular mesenchymal cells (pericytes). In mammals, its architecture involves niches of hematopoietic stem cells, capable of self-regeneration and differentiation into various cell lines.

The concept of niche and adjusting of its microenvironment remain unclear, since the studies were performed basically in vitro^,8,9,10,13,19,30^. Hematopoiesis is of a complex achievement by the differentiation of stem cells, mainly in hematopoietic niches; in normal conditions these cells remain dormant in the bone marrow by the low oxygen concentration, and the proper balance involves chemokines and molecular pathways that are still little understood^14^.

Hematopoiesis occurs along a complex pathway by stem cell differentiation mainly in hematopoietic niches; on normal condition, stem cells remain inactive in bone marrow due to a low oxygen concentration; this balance involves chemokines and molecular pathways still to be fully understood^14^. When changes in bone marrow microenvironment occur, there is releasing of hematopoiesis precursor cells in peripheral blood stream which in spleen find similar condition to develop its cellular lineages. Megakaryocyte in this area can be considered a dysplasia, as there are important cellular morphological changes, with abnormal increase of nucleus with lobule formation^21^.

Hematopoiesis may happen out of bone marrow as long as interaction among systemically factors, microenvironment of a specific tissue and a stem cell niche allows its reactivation, basically in a situation where there is bone marrow failure (myelofibrosis, myelodisplasia, drug toxicity, radiation, infection, neoplasm and autoimmune diseases); significant myelostimulation (necessity for the production of blood cells in inflammatory and hematologic diseases); inflammation and repairing, as it occurs during abnormal chemokine production^13^ ^,^ ^14^. Classically associated to myeloproliferative or myelodisplasic alteration, extramedullary hematopoiesis in spleen related or not to neoplasm disease can arise during reacting situation, although this differentiation might be difficult to be diagnosed without immunohistochemical methods^21^.

When inflammatory process occurs, there can be found next to capillaries mesenchymal undifferentiated cells (pericytes) that are similar to fibroblasts, exhibiting potential of differentiation from other connective cells, as well as resident macrophages. The role of these cells during adverse situation complies multiple intra and extracellular factors that have been extensively studied^6^ ^,^ ^10^ ^,^ ^13^ ^,^ ^28^ ^,^ ^30^. The concept of vascular niche is relatively recent, and it has been associated to bone marrow and spleen sinusoidal endothelium, but the factors involved in its establishment are not well known yet. The activation of multipotent cells, including megakaryocytic precursors involves cells that are adjacent to sinusoidal endothelium to establish and support hematopoietic activity not only in the spleen but in other tissues as well. In neoplasm disease, vascular endothelium works as an extension of bone marrow vascular niches making possible hematopoietic stem cell differentiation in a number of sites. This phenomenon is facilitated by the release of factors by endothelial cells and possible by macrophages. Precursors of hematopoietic cells can also be found in the vessels adjacent to endothelial cells of the spleen, skeletal muscle, small intestine and adipose tissue; many of these tissues are considered extramedullary hematopoiesis sites in adult animals^13^. The continuity of this research aims to assess the perivascular undifferentiated cells once in this experiment neovascularization tissues near the intense material implanted were evidenced.

When extramedullary hematopoiesis secondary to myelostimulation occurs, there is reactivation of embryonic hematopoiesis locations, mainly in the spleen and liver, but sometimes in other tissues, such as a homeostatic natural response in infections, inflammatory diseases, autoimmune and hematological disorders^13^ ^,^ ^14^; this condition has been described as incidental finding on routine histological studies even in spleens removed after traumatic injury^1^ ^,^ ^21^, in biopsies of transplanted livers, after bone marrow transplantation, in thrombocytopenic purpura, hemolytic uremic syndrome, in diseases that accompany hemolysis, splenic infarction, and fibrocongestive splenomegaly^13^ ^,^ ^21^.

In addition to the hemolytic anemia, the hypoxia caused by significant bleeding stimulates the maturation of erythrocyte precursors^13^; in diseases such as sickle cell anemia, thalassemia and hereditary spherocytosis, paravertebral masses may arise of nodular appearance adjacent to the spinal canal, with impairment of thoracic and abdominal cavity^13^ ^,^ ^17^ ^,^ ^18^.

The mouse and the adult rat preserve some hematopoietic activity in spleen and liver; in animals this finding can be considered incidental, but also interpreted as initial indicator of hematologic disease. When there are stimuli to immune response or infections in mammals and rodents, there can be found hematopoiesis in the lungs, kidneys and peritoneal cavity, as well as in the liver and spleen^13^.

The occurrence of neoplasm, both the primary as acute leukemia AML-M7, a rare subtype originated from primitive megakaryocytes^9^, as well as the possible arising of neoplasm lesions associated with chronic infection, represent vast field of research. Studying the histopathology of herniary sac, Barbosa *et al.* ^25^ described the inflammatory infiltrate found in the peritoneum and mesentery, with vascular proliferation of angiomatoid or hamartomatous aspect, large number of monocytes, lymphocytes, macrophages and inappropriate inflammatory reaction, in addition to mesothelial hyperplasia which can be misunderstood with mesothelioma; the authors also criticized the indiscriminate use of synthetic prostheses. Birolini *et al*.^7^ described squamous cell carcinomas along with the chronic infection that can complicate the prosthesis implant in abdominal wall hernia for the repair of incisional hernia, reporting the potential for carcinogenesis in this situation. The niches of megakaryocytes in atypical location highlighted in this experiment can be considered a dysplasia^21^, indicating that the continuation of this study may find interesting results.

There are significant differences that hinder the correct identification of a few cells, which require special staining to be properly highlighted. The dyes used in bone marrow smears are different from H&E of routine histological sections. The bone marrow in response to the continued release of cytokines presents an increase in reticular fibers, which can be highlighted by another classic coloring, picrosirius^11^. Often the immunophenotyping is essential for the differential diagnosis and evaluation of the megakariocytic lineage, since hematopoietic cells contain several antigens; the immunophenotyping by flow cytometry can identify various antigens using specific monoclonal antibodies, which receive the name of “clusters of differentiation” (CD), followed by a specific number. The most important antigens for identification of the megakariocytic lineage are the CD41a, CD42b (megakaryoblast phases) and CD61 (during megakariocytic maturation)^9, 21, 27^, which will be further approached.

Serigiolle *et al.* ^25^ demonstrated that this experimental model of different implant prostheses in hernia defect previously induced, presents a high percentage of adhesions, concluding that it is not the most suitable factor for the assessment of degrees of adhesions. There was an intense and disproportionate inflammatory response to the expected from implanted biomaterials, suggesting that the study should be continued, with evaluation of immunophenotyping and monoclonal antibodies for confirmation of these findings.

The intense neovascularization found adjacent to the implant prosthesis, the large amount of macrophages with phagocyted hemosiderin^27^ in cytoplasm, and perivascular cells, seem to be related to the identification of megakaryocytes niche of in the omentum adhered to the abdominal wall, as well as the presence of megakaryocytes in the spleen, inferring a systemic response, indicate that the parameters analyzed in the experiment should be extended.

## CONCLUSION

All animals studied showed the formation of fibrous adhesion, neovascularization, granuloma, and the presence of mesenchymal cells. Cellular responses were observed to inflammation that, although possible, they were unusual, as the presence of megakaryocytes evidenced in the abdominal wall and the spleen, indicating that subsequent approaches with the use of different techniques, should allow assessing, identifying and quantifying the cell lineage involved.
